# Risk Factors for Worsening of Bone Loss in Patients Newly Diagnosed with Inflammatory Bowel Disease

**DOI:** 10.1155/2022/1498293

**Published:** 2022-04-04

**Authors:** Yi Yin, Xiaofeng Lu, Zhun Li, Song Liu, Lihua Shao, Lei Cao, Rui-Qing Liu, Liang-Yu Huang, Zhen-Xing Zhu, Zhen Guo, Yi Li, Wei-Ming Zhu

**Affiliations:** ^1^Department of General Surgery, Jinling Hospital, Medical School of Nanjing University, No. 305 East Zhongshan Road, Nanjing, China; ^2^Department of General Surgery, Nanjing Drum Tower Hospital, The Affiliated Hospital of Nanjing University Medical School, No. 321 Zhongshan Road, 210008 Nanjing, Jiangsu, China; ^3^Department of Gastrointestinal Surgery, The Affiliated Hospital of Qingdao University, Qingdao, Shandong Province, China

## Abstract

**Background:**

Bone loss is common in patients with inflammatory bowel disease (IBD). The aim of the present study was to determine the prevalence of metabolic bone disease in patients newly diagnosed with IBD and to identify the risk factors for bone loss over time.

**Methods:**

We performed a retrospective, both cross-sectional and longitudinal, study to extract the risk factors of bone loss (including osteopenia and osteoporosis) in patients newly diagnosed with IBD, using dual-energy X-ray absorptiometry (DXA).

**Results:**

A total of 639 patients newly diagnosed with IBD that had at least one DXA were included in the cross-sectional study. Osteopenia and osteoporosis were diagnosed in 24.6% and 5.4% of patients, respectively. Age at diagnosis, body mass index, and serum phosphorus were identified as independent factors associated with bone loss at baseline. A total of 380 of the 639 IBD patients (including 212 CD patients and 168 UC patients) with at least a second DXA scan were included in the longitudinal study. 42.6% of the patients presented a worsening of bone loss in the follow-up study. Menopause, albumin, and use of corticosteroids were identified as independent factors associated with worsening of bone loss.

**Conclusions:**

Metabolic bone disease is common in IBD patients, and there is a significant increase in prevalence of bone loss over time. Postmenopausal female, malnourished patients, and those requiring corticosteroid treatment are at risk for persistent bone loss. Therefore, BMD measurements and early intervention with supplementation of calcium and vitamin D are recommended in IBD patients with high-risk factors.

## 1. Introduction

Inflammatory bowel disease (IBD), including ulcerative colitis (UC) and Crohn's disease (CD), is considered a chronic relapsing autoimmune disease. IBD causes gastrointestinal inflammation and extraintestinal complications such as osteopenia and osteoporosis, characterized by bone loss and low bone mineral density (BMD) [1, 2]. For now, bone loss has become an important clinical problem in patients with IBD [3]. Osteoporosis is present in 13~42% of patients [4], but osteopenia has been reported in up to 77% of patients with IBD [5]. Meanwhile, the occurrence of fracture correspondingly increases by approximately 40%-60% compared to controls, which is associated with significant morbidity and hospitalization [6–8].

The etiology of decreased BMD in patients with IBD appears compromising multiple clinical and genetic backgrounds. The clinical factors leading to low BMD include age, smoking status, corticosteroid use, malnutrition, a history of small bowel resection, disease activity, vitamin D deficiency, low calcium intake, and presence of proinflammatory cytokines [9, 10]. Current guidelines recommend screening for osteoporosis using dual-energy X-ray absorptiometry (DXA) in high-risk IBD patients. Although IBD patients differ in published guidelines, all guidelines agree that DXA scan should be performed in patients with systemic corticosteroid use longer than three months or persistently active disease [1, 11–14].

Of note, with prolonged course of the disease, bone loss aggravated in patients newly diagnosed with IBD [15, 16]. The underlying risk factors contributing to the worsening of bone loss are complex and have not yet been fully revealed. Meanwhile, there are scarce data of BMD in large cohorts from Asian countries. It is inappropriate to apply the factors extracted from Western countries to Asian patients for the differences in genetics, environmental factors, and available treatment agents. Therefore, we aimed to determine the prevalence of bone loss in Chinese patients newly diagnosed with IBD, explore bone mineral density changing trend in the course of the disease, and further extract the risk factors contributing to the worsening of bone loss.

## 2. Patients and Methods

### 2.1. Patient Selection and Study Design

We performed a retrospective, both cross-sectional and longitudinal, study to extract the risk factors for bone loss (including osteopenia and osteoporosis) in patients newly diagnosed with IBD. Patients were identified from the IBD Center of Jinling Hospital. All of the patients included in this study were diagnosed as CD or UC based upon the usual clinical, radiological, endoscopic, and histological criteria [17]. Of note, all patients received DXA scan as a routine exam when they were diagnosed with IBD and during their follow-up. Patients enrolled in this study were those who had at least 1 DXA study after IBD diagnosis between 2010 and 2018. Exclusion criteria were patients with metabolic diseases which might affect BMD, including malabsorption syndrome, chronic kidney disease, diabetes mellitus, abnormal liver function, and a history of corticosteroids or hormonal therapy before diagnosis. BMD expressed only by *Z*-score, time less than 1 year between baseline DXA and the last visit (for the longitudinal study), and neoplasms which led to chemotherapy were also excluded from this study. The Ethics Committee of Jinling Hospital approved the study, and the data analysis was performed according to the Declaration of Helsinki.

### 2.2. Cross-Sectional Study

Baseline characteristics of patients were obtained from their medical chart records, including body mass index (BMI), smoking and drinking habits, symptoms duration, body composition, and biochemical characteristics, as well as Montreal classification for CD and UC. The baseline characteristics of patients were categorized by disease entity (CD and UC), while bone loss was used as the dependent variable in the subsequent univariate and multivariate regression analysis of risk factors for metabolic bone disease.

### 2.3. Longitudinal Study

Patients with at least a second DXA scan (time more than 1 year between baseline and the last DXA scan) were included in the longitudinal study. Besides the baseline characteristics, these parameters were retrieved: years from diagnosis, intestinal surgery, and medical treatment. Supplementation of calcium, vitamin D, and bisphosphonates was also recorded. Active smoking was recorded as consumption more than 7 cigarettes per week, while ex-smokers was recorded as cessation of smoking more than 6 months. Alcohol intake was recorded as consumption of alcohol more than 1 drink per day. Calcium, vitamin D, or bisphosphonate supplementation was recorded as daily supplementation of calcium, vitamin D, or bisphosphonates for more than 6 months up to the time of the last DXA scan. Use of corticosteroids was recorded as cumulative dose of prednisone (to standardize dosing, 0.8 mg methylprednisolone was calculated as 1 mg prednisone). The dose was 1 mg/kg per day (maximum 60 mg per day), and the treatment time was 1 or 2 weeks. Drug withdrawal was a decrease of 10 mg per week until 20 mg per day, and then, the decrease fell to 5 mg per week until total withdrawal. The use of immunomodulators (IMMs), biologics, intestinal surgeries, and other disease-related variables were also included in the follow-up study.

### 2.4. Measurement of BMD

The BMD of the lumbar spine, femoral neck, and total hip were measured in patients newly diagnosed with IBD with dual-energy X-ray absorptiometry (DXA, HITACHI DCS-900FX, Japan). BMD was defined according to the World Health Organization criteria [18]. *T*-score was assessed by the categorization of abnormality in an individual according to the number of standard deviations (SDs) relative to that in young normal controls. For the indication of normal BMD, cutoff of *T* ≥ −1 was used. For the diagnosis of osteopenia and osteoporosis, cutoffs of −2.5 < *T* < −1 and *T* ≤ −2.5 were used, respectively. The lowest score of lumbar spine, femoral neck, or total hip was used for analysis. Bone loss was defined as *T* < −1.0 (including osteopenia and osteoporosis). In the longitudinal study, for those patients who had at least a second DXA, the most recent DXA was retrieved as the control DXA.

### 2.5. Body Composition and Lab Tests

Data of body composition and blood biochemical parameters accessed at the most recent DXA measurement were retrieved from medical chart record. C-reactive protein (CRP), albumin, calcium, and phosphorous were included in the lab tests. BMI was calculated by dividing weight (kg) by the square of height (m). FMI (fat mass index) was calculated by dividing fat mass (kg) by the square of height (m), while ASMI (appendicular skeletal muscle index) was calculated by dividing appendicular skeletal muscle (kg) by the square of height (m). BMI ≤ 18.5 was defined as underweight according to the USA federal guidelines [19].

### 2.6. Statistical Analysis

Clinical characteristics of the patients were compared using the chi-squared test, unpaired Student's *t*-test, and Mann–Whitney *U* test. Univariate and multivariate logistic regression analyses were used to determine potential risk factors of bone loss in patients. For the multivariate models, variables in the univariate analysis with a *p* value ≤ 0.1 were included in a backward strategy, and odds ratios with 95% confidence intervals were estimated. A *p* value < 0.05 was considered statistically significant. All statistical analyses were performed using IBM SPSS Statistics version 25.

## 3. Results

### 3.1. Cross-Sectional Study

#### 3.1.1. Demographic and Clinical Baseline Characteristics

**Figure 1:**
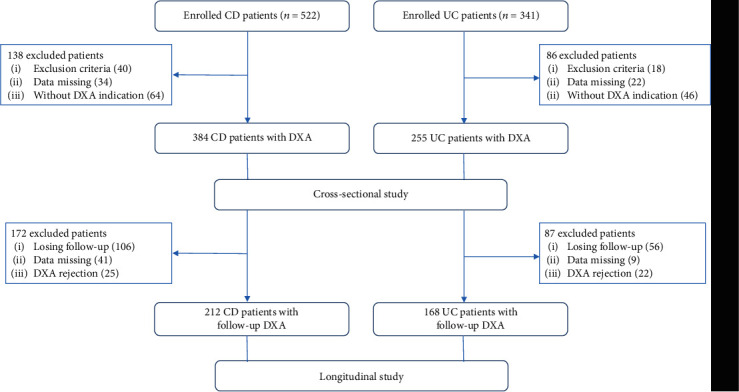
Flowchart of the current study. 639 patients newly diagnosed with inflammatory bowel disease were included in the cross-sectional study, 380 of which completed follow-up DXA and subsequently included in the longitudinal study. Abbreviations: CD: Crohn's disease; UC: ulcerative colitis; DXA: dual-energy X-ray absorptiometry.

A total of 863 IBD patients newly diagnosed with IBD between 2010 and 2018 were retrieved from our database, 639 of which who had at least one DXA were included in the cross-sectional study (Figure 1). The clinical characteristics including baseline data, body composition, biochemical characteristics, and Montreal classification are summarized in Tables 1 and 2. CD was diagnosed in 384 (60.1%) patients and UC in 255 (39.9%), respectively. Mean age (±SD) of the patients in the CD and UC groups was 34.76 ± 9.44 and 35.95 ± 15.14. 52.6% of CD patients and 54.1% UC patients were male. The symptom duration (months) was significantly longer in the UC group than in the CD group (10.68 ± 1.48 and 8.92 ± 1.23, *p* < 0.001), and the smoking population was significantly higher in the CD group than UC (47.4% and 37.6%, *p* = 0.015). With regard to parameters of body composition, the UC group had significantly higher body mass index (BMI, 20.58 ± 3.14 and 20.13 ± 2.56, *p* = 0.048) and appendicular skeleton muscle index (ASMI, 7.81 ± 1.45 and 7.52 ± 1.33, *p* = 0.010) compared to the CD group. In addition, a higher concentration of albumin in plasma was found among CD compared with UC patients (38.72 ± 4.36 and 36.79 ± 5.10, *p* < 0.001), while patients diagnosed with UC had a higher concentration of phosphorus (1.32 ± 0.27 and 1.27 ± 0.25, *p* = 0.017).

#### 3.1.2. BMD and Prevalence of Bone Diseases in IBD Patients

In all cases, mean *T*-score in the femoral neck was −0.8 ± 1.16, that in the lumbar spine was −0.75 ± 1.15, and that in the total hip was −0.73 ± 1.04; in the CD group, mean *T*-score in the femoral neck was −0.82 ± 1.13, that in the lumbar spine was −0.76 ± 1.15, and that in the total hip was −0.74 ± 1.02; in the UC group, mean *T*-score in the femoral neck was −0.76 ± 1.2, that in the lumbar spine was −0.73 ± 1.16, and that in the total hip was −0.72 ± 1.08.

The prevalence of bone diseases, including osteopenia and osteoporosis, in patients with IBD, CD, and UC is demonstrated in Table 3. According to the aforementioned *T*-score criteria, 70% of the patients newly diagnosed with IBD, including 67.4% CD and 73.7% UC, showed normal BMD in all regions (*T*‐score > −1.0). On the contrary, 24.6% of the patients in all cases, including 26.3% in the CD group and 22.0% in the UC group, met the criteria for osteopenia diagnosis (−1.0 > *T*‐score > −2.5) in any region, while 5.4% of the patients in all cases, including 6.3% in the CD group and 4.3% in the UC group, were diagnosed with osteoporosis, respectively. There were no significant differences between the UC and CD groups in the occurrence of metabolic bone diseases, although the prevalence was slightly higher in the CD group.

#### 3.1.3. Univariate and Multivariate Analysis of Risk Factors for Bone Loss in Patients Newly Diagnosed with IBD

Univariate and multivariate (regression) analysis for identification of risk factors for bone loss in patients newly diagnosed with IBD was performed for one dependent variable: bone loss (including osteopenia and osteoporosis). Univariate variables that had a *p* value ≤ 0.1 were all entered in a multivariate logistic regression model including age at diagnosis, BMI, FMI, and phosphorus for all cases (IBD); age at diagnosis, BMI, alcohol intake, and phosphorus for the CD group; and age at diagnosis, BMI, and phosphorus for the UC group. We used a backward strategy and abandoned all the less significant variables in each step. Finally, all the relevant independent risk factors and protective factors were identified and their odds ratio (OR), 95% confidential intervals (95% CI), and *p* value are demonstrated in Table 4.

It was revealed that for patients newly diagnosed with IBD, increasing age at diagnosis was identified as the risk factor for bone loss; increasing BMI and phosphorus were identified as protective factors. For patients newly diagnosed with CD, increasing BMI and phosphorus were identified as protective factors for bone loss. For patients newly diagnosed with UC, increasing age at diagnosis was identified as the risk factor for bone loss and increasing BMI was identified as the protective factor for bone loss. Conversely, the other variables studied were not associated with the occurrence of low BMD.

### 3.2. Longitudinal Study

#### 3.2.1. Baseline Characteristics of the Cohort

380 of the 639 IBD patients (including 212 CD patients and 168 UC patients) with at least a second DXA scan were included in the longitudinal study (Figure 1). Besides the baseline characteristics, the subsequent medical treatment, including hospitalization, intestinal surgery, and use of corticosteroids, IMM, and biologics, as well as supplementation of calcium, vitamin D, or bisphosphonates were assessed and analyzed (Table 5). The worsening of bone loss was defined as from normal to osteopenia or osteoporosis and from osteopenia to osteoporosis. Median interval between the first and last DXA was 3.52 ± 2.33 years. A total of 86.5% IBD patients received corticosteroids, IMM, or biologics, while 46.3% of which required treatment intensification (adding an immunomodulatory, a biologic or increasing biologic dose) during follow-up. A total of 62.7% IBD patients received calcium plus vitamin D treatment, while 18.5% of which were treated with bisphosphonate supplementation.

**Figure 2:**
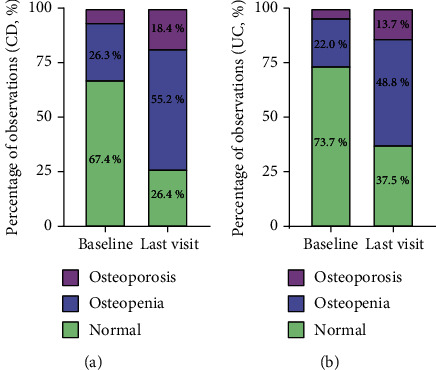
Difference between the proportion of (a) CD and (b) UC patients with osteopenia and osteoporosis at the baseline and at the last visit. Abbreviation: CD: Crohn's disease; UC: ulcerative colitis.

162 (42.6%) of the 380 patients were included in the follow-up study who presented normal values or osteopenia in the baseline DXA that had a worsening in the control DXA. As is shown in Figure 2, in the CD group, patients with osteopenia increased from 26.3% to 55.2% (*p* < 0.001), while patients with osteoporosis increased from 6.3% to 18.4% (*p* < 0.001). In the UC group, patients with osteopenia increased from 22.0% to 48.8% (*p* < 0.001), while patients with osteoporosis increased from 4.3% to 13.7% (*p* = 0.001). These results demonstrate that a fair number of newly diagnosed IBD patients underwent constant bone loss during the course of the disease. The risk factors associated with the persistent bone loss were identified in the subsequent multivariate analysis.

#### 3.2.2. Univariate and Multivariate Analysis of Risk Factors for Worsening of Bone Loss in Patients with IBD

Univariate and multivariate (regression) analysis for extraction of risk factors for worsening of bone loss was performed for one dependent variable: worsening of bone loss (from normal to osteopenia or osteoporosis and from osteopenia to osteoporosis). Univariate variables that had a *p* value ≤ 0.1 were all entered in a multivariate logistic regression model including menopause, intestinal surgery, albumin, phosphorus, corticosteroids, calcium, and vitamin D for all cases (IBD); BMI, menopause, CRP, albumin, and biologics for the CD group; and smoking, menopause, intestinal surgery, FMI, albumin, corticosteroids, calcium, and vitamin D for the UC group. All the relevant independent risk factors and protective factors were identified, and their odds ratio (OR), 95% confidential intervals (95% CI), and *p* value are demonstrated in Table 5. It was revealed that, for IBD patients, menopause and use of corticosteroids were identified as risk factors for worsening of bone loss; increasing albumin was identified as the protective factor. For CD patients, menopause was identified as the risk factor for constant bone loss, while increasing albumin was identified as the protective factor. For UC patients, menopause and corticosteroids were identified as risk factors for worsening of bone loss.

## 4. Discussion

The present study describes the largest Chinese cohort of IBD patients in whom bone mineral density is analyzed. To determine the risk factors of bone loss in patients newly diagnosed with IBD and the worsening of bone loss during disease course, an observational, retrospective, both cross-sectional and longitudinal study was conducted. It was revealed that the prevalence of osteopenia and osteoporosis in Chinese patients newly diagnosed with IBD was 24.6% and 5.4%. The clinical risk factors for bone loss in patients newly diagnosed with IBD were age at diagnosis, BMI, and phosphorus. Of note, there was a marked increase in the prevalence of osteopenia and osteoporosis with disease progressing. CD patients with bone loss increased from 32.6% to 73.6%, while UC patients with bone loss increased from 26.3% to 62.5%. We have identified menopause, albumin, and use of corticosteroids as risk factors for the worsening of bone loss in IBD patients.

Metabolic bone disease (including osteopenia and osteoporosis) is a well-recognized complication in IBD patients, characterized by reduced bone mineral density and higher risk of fractures [7, 20]. The prevalence of osteoporosis in China varies based on age, gender, and bone site measurements in the general population [21, 22]. Its prevalence among men and women older than 50 years ranges from 10.1% to 19.2% and from 14.2% to 33.8%, respectively. However, the prevalence dramatically decreases in the younger population. In a Chinese population-based study, it was revealed that in men younger than 50 years, the prevalence of osteoporosis was 0.4% when femoral neck was measured and 2.1% when accessed with lumbar spine [22]. In the present study, the prevalence of osteoporosis in patients newly diagnosed with IBD is similar with that of the same age group in Chinese general population, which was also consistent with previous studies [15, 16]. It has been reported that low BMD was more common in patients newly diagnosed with CD rather than UC [23, 24]. From our study, the prevalence of osteoporosis in CD patients was slightly higher than UC patients, although the difference was not significant.

Relative high BMI has long been regarded to protect against osteoporosis and fragility fractures in the general population [25–27]. Meanwhile, BMI was also a major clinical determinant, demonstrating a negative correlation with BMD in all IBD patients. We identified low BMI as an independent risk factor for low BMD both in CD and UC patients, which was in agreement with previous studies [28–30]. As for patients newly diagnosed with IBD, it is difficult to conclude that whether low BMI is a risk factor for bone loss or merely a marker of disease severity from the cross-sectional study. Age and menopause are widely regarded as risk factors for bone loss either in general population [21] or in IBD patients [31]. In elderly patients, their BMD can be influenced by the status of sex hormones, decreased opportunities for weight-bearing exercise, comorbidities, and drug use. In children and young adults with IBD, other factors such as disease duration, height, cumulative duration of steroid therapy, physical activity, and nasogastric tube feeding were associated with BMD [32].

Of note, we noticed marked increased prevalence of metabolic bone disease over time in patients newly diagnosed with IBD. IBD patients are more susceptible to bone loss due to corticosteroid treatment, hospitalizations, nutritional deficiencies, and systemic inflammation [5, 33]. As aforementioned, current guidelines published by the British Society of Gastroenterology (BSG), the American Gastroenterological Association (AGA), and European Crohn's and Colitis Organization (ECCO) all agree that patients with systemic corticosteroid use longer than three months or persistently active disease are high-risk patients for osteoporosis. As suggested by guidelines, calcium and vitamin D supplementation was added in the daily treatment in high-risk patients, which seems to be a protective factor for worsening of bone loss in the univariate analysis (OR = 0.636, CI 95% = 0.425–0.879, *p* = 0.014), although this association disappears when the elected variables are included in the model. Moreover, fat mass index (FMI) was associated with osteoporosis in our univariate analysis, but multivariate analysis failed to confirm this beneficial effect in cross-sectional study. Both fat tissue and lean tissue showed positive relationships with BMD, and lean tissue showed a much stronger correlation than fat tissue according to previous studies [10, 27].

Moreover, surgery is also a significant factor associated with bone loss in patients with IBD. Osteoporosis is common in UC patients who have undergone restorative proctocolectomy with pouch-anal anastomosis (IPAA) [34, 35] and in CD patients after bowel resection and creation of stoma [36, 37]. Prevalence of bone loss in patients with intestinal surgery may be associated with malabsorption, dysbiosis of gut microbiome, and SCFA deficiency. We also identified intestinal surgery as a risk factor for worsening of bone loss in the longitudinal study, especially in UC patients, although the difference was not significant in the multivariate study which may be associated with a limited sample size.

There are several limitations for this study. First, this is a retrospective study, from a single center, with potential bias due to exclusion of patients with missing clinical data. Second, although indirect measures of IBD activity such as use of corticosteroids and intestinal surgery were included, it is hard for us to extract reliable IBD activity data (standardized clinical and endoscopic scores) from the chart review. Finally, no control group of non-IBD patients is available which prevents us to specifically determine the increase in osteoporosis risk attributable to IBD.

In conclusion, the present study demonstrates that metabolic bone disease is common in patients newly diagnosed with IBD. Of note, there is a significant increase in prevalence of bone loss over time. Clinicians should be aware that postmenopausal female, malnourished patients, and those requiring corticosteroid treatment are at risk for constant bone loss. Therefore, BMD measurements and early intervention with supplementation of calcium and vitamin D are supposed to be carried out in IBD patients with high-risk factors.

## Figures and Tables

**Table 1:** Clinical characteristics.

Characteristics	IBD	CD	UC	*p*
Number of patients	639	384	255	
Age at diagnosis (year)	35.23 ± 12.04	34.76 ± 9.44	35.95 ± 15.14	0.405
Male sex, *n* (%)	340 (53.2)	202 (52.6)	138 (54.1)	0.707
Smoking, *n* (%)	278 (43.5)	182 (47.4)	96 (37.6)	0.015
Ex-smokers	45 (16.8)	17 (9.9)	28 (29.2)	
Active	223 (83.2)	155 (90.1)	68 (70.8)	
Alcohol intake, *n* (%)	233 (36.5)	131 (34.1)	102 (40)	0.121
Symptoms duration (months)	9.62 ± 1.59	8.92 ± 1.23	10.68 ± 1.48	<0.001
Menopause, *n* (%)	43 (14.4)	25 (13.7)	18 (15.4)	0.692
Body composition				
BMI (kg/m^2^)	20.31 ± 2.81	20.13 ± 2.56	20.58 ± 3.14	0.048
FMI (kg/height m^2^)	9.17 ± 2.67	9.23 ± 2.86	9.08 ± 2.35	0.487
ASMI (kg/height m^2^)	7.64 ± 1.39	7.52 ± 1.33	7.81 ± 1.45	0.010
Biochemical characteristics				
CRP (mg/L)	15.67 ± 28.18	14.58 ± 25.22	17.32 ± 32.12	0.229
Albumin (g/L)	37.95 ± 4.76	38.72 ± 4.36	36.79 ± 5.10	<0.001
Calcium (mmol/L)	2.25 ± 0.18	2.24 ± 0.16	2.26 ± 0.21	0.173
Phosphorus (mmol/L)	1.29 ± 0.26	1.27 ± 0.25	1.32 ± 0.27	0.017

Data are presented as means ± standard deviation (range) or *n* (%). Abbreviations: BMI: body mass index; FMI: fat mass index; ASMI: appendicular skeletal muscle index; CRP: C-reactive protein.

**Table 2:** Montreal classification.

Crohn's disease, *n* (%)	384 (60.1)
Age	
A1 (≤16)	55 (14.3)
A2 (17-39)	212 (55.2)
A3 (≥40)	117 (30.5)
Location	
L1 (ileal)	114 (29.7)
L2 (colonic)	103 (26.8)
L3 (ileocolonic)	167 (43.5)
+L4 (upper disease)	6 (1.6)
Behaviour	
B1 (nonstricturing, nonpenetrating)	268 (69.8)
B2 (stricturing)	59 (15.4)
B3 (penetrating)	57 (14.8)
+p (perianal disease)	86 (22.4)
Ulcerative colitis, *n* (%)	255 (39.9)
Proctitis	45 (17.6)
Left-sided colitis	89 (34.9)
Extensive colitis	121 (47.5)

**Table 3:** Prevalence of bone diseases in patients according to *T*-scores.

	IBD	CD	UC	*p*
Number of patients	639	384	255	
All regions normal (*T*‐score > −1.0)	447 (70)	259 (67.4)	188 (73.7)	0.090
Osteopenia (−1.0 > *T*‐score > −2.5)	157 (24.6)	101 (26.3)	56 (22.0)	0.212
Neck	75 (11.7)	53 (13.8)	22 (8.6)	0.047
Spine	42 (6.6)	27 (7.0)	15 (5.9)	0.566
Hip	40 (6.3)	21 (5.5)	19 (7.5)	0.311
Osteoporosis (*T*‐score < −2.5)	35 (5.4)	24 (6.3)	11 (4.3)	0.292
Neck	15 (2.3)	12 (3.1)	3 (1.2)	0.091
Spine	13 (2.0)	8 (2.1)	5 (2.0)	0.914
Hip	7 (1.1)	4 (1.0)	3 (1.2)	0.873

Abbreviations: IBD: inflammatory bowel disease; CD: Crohn's disease; UC: ulcerative colitis.

**Table 4:** Univariate and multivariate analysis of risk factors for bone loss in patients newly diagnosed with inflammatory bowel disease.

	IBD			CD			UC		
	Univariate	Multivariate		Univariate	Multivariate		Univariate	Multivariate	
	*p*	*p*	OR (95% CI)	*p*	*p*	OR (95% CI)	*p*	*p*	OR (95% CI)
Disease type	0.658			NA	NA		NA	NA	
Age at diagnosis (year)	0.023	0.037	2.320 (1.155–4.854)	0.056	0.23	1.003 (0.703–1.432)	0.036	0.043	4.896 (1.223–18.607)
Sex	0.382			0.689			0.244		
BMI (kg/m^2^)	<0.001	0.005	0.373 (0.206–0.692)	0.004	0.022	0.573 (0.348–0.862)	<0.001	0.002	0.445 (0.263–0.765)
Smoking	0.102			0.236			0.754		
Alcohol intake	0.512			0.059	0.836	0.974 (0.760–1.249)	0.343		
Symptom duration (months)	0.879			0.939			0.623		
Menopause	0.760			0.865			0.136		
Hospitalizations	0.389			0.454			0.613		
Intestinal surgery history	0.864			0.882			0.471		
Body composition									
FMI (kg/height m^2^)	0.056	0.408	0.902 (0.653–1.247)	0.209			0.142		
ASMI (kg/height m^2^)	0.127			0.354			0.481		
Biochemical characteristics									
CRP (mg/L)	0.778			0.403			0.914		
Albumin (g/L)	0.403			0.318			0.632		
Calcium (mmol/L)	0.133	0.673		0.219			0.345		
Phosphorus (mmol/L)	0.089	0.018	0.562 (0.274–0.831)	0.026	0.003	0.456 (0.241–0.864)	0.131	0.654	0.872 (0.585–1.310)

Abbreviations: IBD: inflammatory bowel disease; CD: Crohn's disease; UC: ulcerative colitis; BMI: body mass index; FMI: fat mass index; ASMI: appendicular skeletal muscle index; CRP: C-reactive protein; OR: odds ratio; CI: confidence interval.

**Table 5:** Univariate and multivariate analysis of risk factors associated with worsening of bone loss in patients with IBD during follow-up.

	IBD			CD			UC		
	Univariate	Multivariate		Univariate	Multivariate		Univariate	Multivariate	
	*p*	*p*	OR (95% CI)	*p*	*p*	OR (95% CI)	*p*	*p*	OR (95% CI)
Disease type	0.124			NA	NA		NA	NA	
Age at diagnosis (year)	0.689			0.823			0.776		
Disease duration (year)	0.372			0.126			0.414		
Sex	0.871			0.769			0.912		
BMI (kg/m^2^)	0.156			0.032	0.411	0.872 (0.683–1.306)	0.753		
Smoking	0.536			0.453			0.055	0.483	1.012 (0.996–1.032)
Alcohol intake	0.477			0.232			0.689		
Menopause	0.001	<0.001	8.532 (3.356–55.633)	0.023	0.006	5.368 (2.672–39.636)	0.012	0.004	8.016 (4.522–64.326)
Hospitalizations	0.644			0.489			0.572		
Intestinal surgery	0.034	0.088	1.19 (0.986–1.445)	0.268			0.072	0.381	1.621 (0.552–4.786)
Body composition									
FMI (kg/height m^2^)	0.163			0.468			0.062	0.353	0.67 (0.27–1.64)
ASMI (kg/height m^2^)	0.671			0.244			0.893		
Biochemical characteristics									
CRP (mg/L)	0.684			0.032	0.357	0.957 (0.895–1.023)	0.843		
Albumin (g/L)	0.022	0.036	0.679 (0.521–0.862)	0.014	0.034	0.664 (0.528–0.833)	0.074	0.233	0.988 (0.963–1.013)
Calcium (mmol/L)	0.868			0.904			0.561		
Phosphorus (mmol/L)	0.072	0.689	0.993 (0.979–1.008)	0.655			0.243		
Concurrent status									
Short bowel syndrome	0.587			0.664			0.926		
Stoma	0.315			0.264			0.547		
Medical treatment									
Corticosteroids	0.176	0.012	2.73 (1.143–6.236)	0.728			0.089	0.006	2.308 (1.659–4.632)
IMMs	0.284			0.517			0.475		
Biologics	0.362			0.108	0.082	0.611 (0.284–1.315)	0.469		
Calcium and vitamin D	0.014	0.259	0.98 (0.827–1.155)	0.688			0.035	0.451	0.994 (0.987–1.002)
Bisphosphonates	0.861			0.939			0.542		

Abbreviations: IBD: inflammatory bowel disease; CD: Crohn's disease; UC: ulcerative colitis; BMI: body mass index; FMI: fat mass index; ASMI: appendicular skeletal muscle index; CRP: C-reactive protein; IMMs: immunomodulators; OR: odds ratio; CI: confidence interval.

## Data Availability

The data used to support the findings of this study were supplied by Yan Zhou (Big Data Center, Nanjing General Hospital of Military Command) under license and so cannot be made freely available. Requests for access to these data should be made to Yi Yin, 18795891071@163.com.
